# Direction of Arrival Estimation Using Two Hydrophones: Frequency Diversity Technique for Passive Sonar

**DOI:** 10.3390/s19092001

**Published:** 2019-04-29

**Authors:** Peng Li, Xinhua Zhang, Wenlong Zhang

**Affiliations:** 1 Acoustic Science and Technology Laboratory, Harbin Engineering University, Harbin 150001, China; xinhua_zh@126.com; 2 Key Laboratory of Marine Information Acquisition and Security (Harbin Engineering University), Ministry of Industry and Information Technology, Harbin 150001, China; 3 College of Underwater Acoustic Engineering, Harbin Engineering University, Harbin 150001, China; 4 Department of Underwater Weaponry & Chemical Defense, Dalian Navy Academy, Dalian 116018, China; 15604285615m0@sina.cn

**Keywords:** direction of arrival estimation, frequency diversity, passive sonar

## Abstract

The traditional passive azimuth estimation algorithm using two hydrophones, such as cross-correlation time-delay estimation and cross-spectral phase estimation, requires a high signal-to-noise ratio (SNR) to ensure the clarity of the estimated target trajectory. This paper proposes an algorithm to apply the frequency diversity technique to passive azimuth estimation. The algorithm also uses two hydrophones but can obtain clear trajectories at a lower SNR. Firstly, the initial phase of the signal at different frequencies is removed by calculating the cross-spectral density matrix. Then, phase information between frequencies is used for beamforming. In this way, the frequency dimension information is used to improve the signal processing gain. This paper theoretically analyzes the resolution and processing gain of the algorithm. The simulation results show that the proposed algorithm can estimate the target azimuth robustly under the conditions of a single target (SNR = −16 dB) and multiple targets (SNR = −10 dB), while the cross-correlation algorithm cannot. Finally, the algorithm is tested by the swell96 data and the South Sea experimental data. When dealing with rich frequency signals, the performance of the algorithm using two hydrophones is even better than that of the conventional broadband beamforming of the 64-element array. This further validates the effectiveness and advantages of the algorithm.

## 1. Introduction

Azimuth estimation is an important research area in passive sonar applications. Since two hydrophones are easy to deploy and throwing buoys is also easy in actual combat, azimuth estimation algorithms, based on the cross-correlation time-delay estimation of two hydrophones are often applied to buoys and autonomous underwater vehicles (AUVs) [1]. However, the cross-correlation algorithm requires a high signal-to-noise ratio (SNR) and can only estimate one target. Using only two sensors to estimate more targets and obtain more accurate estimation results has always been the focus of the research on passive sonar applications. 

In passive detection research, many array processing algorithms can improve the performance of azimuth estimation, such as the split aperture method, which can obtain an extremely small size and spacing of the array elements, while avoiding the formation of grating lobes [2], and can also modify the beamforming process, according to the linear phase relationship between two subarrays, to obtain high-precision azimuth estimation results [3]. In addition, the co-prime array algorithm can achieve a higher degree of freedom, with a limited number of elements, thus increasing the number of estimable sources [4]. However, such algorithms need a special array structure first. For example, the split aperture method requires two sub-arrays, with element spacing of (p−1)λ/2 and pλ/2, and co-prime arrays, needing two sub-arrays, have *M* and *N* sensors, where *M* and *N* are co-prime with the appropriate inter-element spacing [5]. For two hydrophones, it is difficult to obtain such spatial information. Therefore, in order to improve the azimuth estimation performance of two hydrophones, only additional information from other dimensions or equivalent spatial information from other dimensions can be added.

In the application of a multiple-input multiple-output (MIMO) radar, there is a frequency diversity array (FDA) technique [6], the idea of which is to combine the spatial information and the frequency information. In 2006, Antonik et al. first proposed the concept of FDA at the International Radar Conference [7]. The algorithm introduces a frequency difference between each array element at the transmitting terminal and combines the distance and the scanning angle to improve the anti-interference ability [8]. In recent years, scholars from various countries have done a great deal of research on FDA, such as improving the practicality of FDA [9], reducing the array cost [10], extending FDA to distance dimensions [11], and applying FDA to the bistatic joint estimation of the distance and azimuth [12]. The application of the FDA algorithm in radars has matured. Researchers have made a comprehensive analysis of the algorithm’s performance [10,11,13]. Whatever the improvement of the algorithm, it is always the case that the phase difference changes, caused by the sound path and the frequency, are used to relate the distance and the angle change. 

In passive sonar applications, the target is often a broadband source. However, the conventional towed array processing only divides the frequency band into many sub-bands. Then, the azimuth estimation results are calculated and added together. Both the wideband processing method [14] and the time domain beamforming algorithm [15] do not take advantage of the relationship between the frequency, target azimuth and signal phase. Inspired by the FDA technique in radars, this paper applies the idea of frequency diversity to the azimuth estimation of two hydrophones in a passive sonar. The information dimension of the dual-element output signal is improved by the frequency information, thereby realizing a high performance of the azimuth estimation. However, the passive algorithm of the two hydrophones has an important difference from the commonly used algorithm in the MIMO radar. That is, the received signal of the passive sonar is unpredictable, and the initial phase of each frequency point is unknown. Therefore, the algorithm first removes the initial phase on each frequency component of the signal by conjugate processing, which calculates the cross-spectral density between the two elements. A frequency domain vector that can be used for beamforming is constructed using a cross-spectrum, the phase of which changes with the target azimuth and the sensor interval between the different frequency. The corresponding weighted vector is designed to obtain the azimuth estimation result.

The remainder of this paper is organized as follows: In Section 2, we first briefly introduce the cross-correlation method, cross-spectral method and FDA. Then the passive azimuth estimation algorithm of two hydrophones, based on the FDA technique, is proposed, and the processing gain and resolution of the algorithm are analyzed. In Section 3, the algorithm and the traditional algorithm are compared by simulation experiments, and the effectiveness and advantages of the algorithm are verified. Section 4, experimental data processing further proves that the proposed two-hydrophone passive azimuth estimation algorithm using the FDA technique is better than the cross-correlation method and can obtain a clear azimuth history diagram. In addition, the influence of the energy spectrum distribution of the signal on the estimation result is analyzed. The final conclusion is given in Section 5. 

## 2. Theoretical Derivation

This paper is based on the idea of FDA technology and proposes a passive azimuth estimation algorithm applied to two hydrophones. First, in Section 2.1, we briefly review the passive azimuth estimation algorithms commonly used in two hydrophones and the frequency diversity techniques used in radar. The algorithm proposed in this paper is introduced in Section 2.2. The resolution and processing gain of the algorithm are analyzed, and the algorithm is extended.

### 2.1. Conventional Algorithm

Section 2.1.1 briefly introduces two commonly used azimuth estimation algorithms on two hydrophones: The cross-correlation method and cross-spectrum method. In Section 2.1.2, the FDA algorithm in a MIMO radar is briefly introduced.

#### 2.1.1. Cross-Correlation Method and Cross-Spectral Method

**Figure 1:**
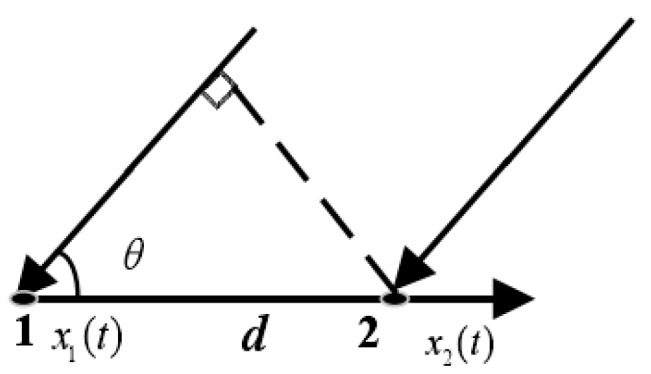
Received signal model using two hydrophones.

First, the cross-correlation method is introduced [16]: the two-hydrophone receiver model is shown in Figure 1, where *d* is the array element spacing and *θ* is the signal incoming wave direction.

$x_{1}(t)$, $x_{2}(t)$ are the received signals of hydrophone 1 and hydrophone 2, respectively, and their cross-correlation functions can be expressed as:

(1) $$R_{x_{1}x_{2}}(\tau )=E[x_{1}(t)x_{2}(t-\tau )]$$

where E[•] is a mathematical expectation. When the noise and the signals are independent of each other, and the SNR is high enough, after calculating the delay $\tau_{0}$, corresponding to the correlation peak, the direction of arrival (DOA) estimation can be acquired, according to Equation (2):

(2) $$\tau_{0}=d\cos\theta /c$$

where *c* is the speed of sound in water. In addition to the cross-correlation delay estimation algorithm, the commonly used algorithm also has a cross-spectral method [17]. Let the Fourier transform of $x_{1}(t)$ be $X_{1}(f)$, and the Fourier transform of $x_{2}(t)$ can be obtained as $X_{1}(f)e^{j2\pi f\tau_{0}}$, according to the delay characteristic of the Fourier transform. Then, the cross-spectrum of hydrophone 1 and the hydrophone 2 can be obtained as follows:

(3) $$Z_{X}(f)=X_{1}{}^{*}(f)X_{2}(f)={\left|X_{1}(f)\right|}^{2}e^{j2\pi f\tau }$$

It can be found, from Equation (3) that the time delay $\tau_{0}$ is included in the phase information of the cross-spectrum, namely:

(4) 2πfdcosθ/c=arctan{Im[Z(f)]Re[Z(f)]}

The DOA can be estimated according to Equation (4). However, such algorithms first have requirements on SNR concerning the received array signals. Secondly, for wideband signals, when using cross-correlation time delay estimation, the cross-correlation function graph shows many periodic peaks [18], which further increases the difficulty of peak finding. Therefore, implementing DOA estimation based on two hydrophones at a low SNR is very important. We found that neither of these algorithms effectively utilized the phase relationship between the frequencies. The FDA technique in a MIMO radar will be described below, which effectively utilizes the phase relationship between the frequencies.

#### 2.1.2. FDA Technique

**Figure 2:**
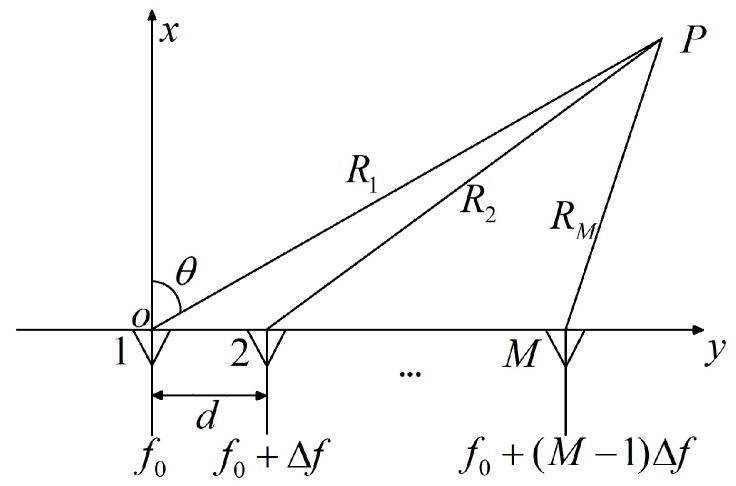
Schematic diagram of an FDA space structure.

As shown in the Figure 2, the frequency of the waveform radiated from each sensor was incremented by Δf from element to element. 

By means of quadrature modulation or matched filtering, only the corresponding frequency signal is received. It is easy to obtain the phase difference between the adjacent elements (*m*th and *m+1*th), which can be written as:

(5) $$\Delta \varphi =2\pi f_{0}d\sin\theta /c-2\pi R_{m}\Delta f/c+2\pi \Delta fd\sin\theta /c.$$

where $R_{m}$ is the distance from the sound source to the *m*th sensor. When the far field condition is met, $R_{m}$ can be recorded as:

(6) $$R_{m}=R_{1}-d\sin\theta .$$

According to the beamforming principle [19], it can be calculated that the phase shift of Equation (5) cause the beam at some apparent angle $\theta^{\prime }$ [20]:

(7) $$\theta^{\prime }=\arcsin\left\{\sin\theta -\frac{R_{1}\Delta f}{df_{0}}+\frac{\Delta f\sin\theta }{f_{0}}\right\}.$$

Equation (7) associates the scan angle $\theta^{\prime }$, the target azimuth θ and the target distance. Therefore, FDA can estimate the DOA and distance and can also suppress clutter interference.

It should be noted that the DOA estimation of the MIMO radar and two passive hydrophones have two important differences: (1) When FDA is applied in the MIMO radar, it is used for multiple array elements, and the frequency of the transmitted signal varies with the number of elements. In the algorithm of this paper, only two array elements are used, and the received signal is sampled in the frequency domain. (2) In the MIMO radar, the waveform of each transmitted signal is known. Therefore, the initial phase of the received signal for each element at each frequency is controllable. In the algorithm of this paper, since it is applied to a passive sonar, the initial phase of each frequency is unknown. Therefore, the application of the idea of FDA to the DOA estimation of two passive hydrophones has to be greatly changed.

### 2.2. FDA Technique of Two Hydrophones

#### 2.2.1. Theory

According to the model in Figure 1, the frequency domain expressions of the received signals of the two hydrophones are:

(8) $$\begin{array}{l} S_{1}(f)=\left|X_{1}(f)\right|e^{j[2\pi fr/c+\phi (f)]}+N_{1}(f)\\ S_{2}(f)=\left|X_{1}(f)\right|e^{j[2\pi fd\cos\theta /c+2\pi fr/c+\phi (f)]}+N_{2}(f) \end{array}$$

where $N_{1}(f)$ and $N_{2}(f)$ are the ambient noise received by hydrophone 1 and hydrophone 2, respectively. ϕ(f) is the random, frequency-dependent phase of the source. It can be observed, from the above equation, that in the phase information of $S_{2}(f)$, the first item contains the azimuth information of the target, the second term relates to the propagation distance, and the third term is the initial phase of the frequency. Since, in the application condition of the passive sonar, the target distance and the initial phase of the sound source signal are unknown, we first calculate the cross-spectrum of the two sensor signals to remove the phase in the second and third terms:

(9) $$Z(f)=S_{1}{}^{*}(f)S_{2}(f)=Z_{X}(f)+Z_{N}(f)$$

where $Z_{X}(f)$ is the cross-spectrum of the signal, and $Z_{N}(f)$ is the component related to environmental noise. $Z_{X}(f)$ and $Z_{N}(f)$ are denoted as:

(10) $$\begin{array}{l} Z_{X}(f)={\left|X_{1}(f)\right|}^{2}e^{j2\pi fd\cos\theta /c}\\ Z_{N}(f)=X_{1}{}^{*}(f)N_{2}(f)+N_{1}{}^{*}(f)X_{2}(f)+N_{1}{}^{*}(f)N_{2}(f) \end{array}$$

After obtaining the cross-spectrum Z(f), $Z(f_{m})$ is obtained by sampling Z(f) in the frequency domain. According to the idea of the frequency diversity technique, the frequency of the sampling point is $f_{m}$, and the frequency increment is Δf. Vector $\left[Z(f_{1}),Z(f_{2}),\ldots ,Z(f_{M})\right]$ can be generated as:

(11) $$Z(f_{m})={\left|X_{1}(f_{m})\right|}^{2}e^{j2\pi f_{m}d\cos\theta /c}+Z_{N}(f),f_{m}=f_{1}+(m-1)\Delta f.$$

It can be found, from Equation (11), that the phase difference of $Z_{X}(f_{m})$ between the adjacent sampling points is j2πΔfdcosθ/c. There is no phase relationship between the various frequencies of ambient noise. Array manifolds are generated by the phase relationship in Equation (12):

(12) $$A_{f_{m}}=e^{-j2\pi m\Delta fd\cos\theta /c}.$$

The beamforming output can be obtained according to the principle of in-phase superposition:

(13) $$\mathrm{Beam}(\theta )=\sum_{m=1}^{M}Z(f_{m})A_{f_{m}}$$

#### 2.2.2. Performance Analysis

According to the above analysis, it is easy to obtain the directivity function of the passive two-hydrophone algorithm based on FDA technology:

(14) $$R\left(\theta \right)=\left|\frac{\sin\left(\pi M\Delta f\frac{d\sin\theta }{c}\right)}{M\sin\left(\pi \Delta f\frac{d\sin\theta }{c}\right)}\right|.$$

The azimuth resolution, based on half the width of the main lobe, is defined as in Equation (14):

(15) $$\theta_{r}=\arcsin(\frac{c}{N\Delta fd}).$$

In order not to obtain a grating lobe, the scanning angle θ needs to satisfy $\sin(\theta )\leq \frac{c}{2\Delta fd}$. In general, the scanning angle is −90° to 90°, so when frequency domain sampling is performed on the signal, the frequency interval $\Delta f\leq \frac{c}{2d}$ should be satisfied.

According to the beamforming of Equation (13), the output SNR is:

(16) $$SNR_{out}=10\log\frac{\sum_{m=1}^{M}{\left|X_{1}(f_{m})\right|}^{2}}{\sum_{m=1}^{M}A_{f_{m}}Z_{N}(f_{m})}.$$

It can be seen from Equation (16) that the less related the ambient noise between the frequencies, the higher the output SNR.

#### 2.2.3. Algorithm Extension: Three Hydrophones

**Figure 3:**
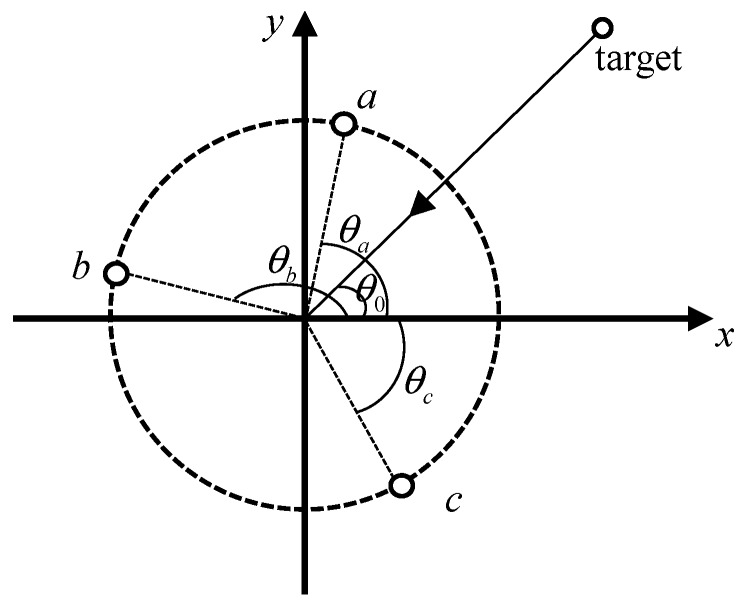
Schematic diagram of the spatial structure of the three hydrophone.

From the above theoretical derivation, we can find that the passive two-hydrophone algorithm based on FDA technology is similar to the single-frequency signal processing algorithm of a conventional towed-line array. Similarly, the algorithm can be extended to higher dimensions, for instance, using a wideband signal to obtain a performance similar to the single-frequency processing of a circular array. The specific process is as follows:

Assume that the three hydrophones, a, b, and c, have a radius of *R*. The angle with the reference abscissa are $\theta_{a}$, $\theta_{b}$ and $\theta_{c}$, respectively. The incident angle of the far-field sound source is $\theta_{0}$. According to the spatial structure of the three hydrophones, as shown in Figure 3, the frequency domain model of the received signals can be obtained as follows:

(17) $$\begin{array}{l} S_{a}(f)=e^{j2\pi f(-R\cos(\theta_{a}-\theta_{0})+r)/c+\varphi (f)}\\ S_{b}(f)=e^{j2\pi f(-R\cos(\theta_{b}-\theta_{0})+r)/c+\varphi (f)}\\ S_{c}(f)=e^{j2\pi f(-R\cos(\theta_{c}-\theta_{0})+r)/c+\varphi (f)} \end{array}$$

where *r* is the propagation distance. Similarly, we also calculate the cross-spectrum to remove the initial phase:

(18) $$\begin{array}{l} Z_{ab}(f_{1})=S_{a}(f_{1})\cdot S_{b}{}^{*}(f_{1})\\ Z_{ac}(f_{m})=S_{a}(f_{m})\cdot S_{c}{}^{*}(f_{m}),\;m=1,\;2,\;3,\;\ldots ,\;M \end{array}$$

where *f*_1_ is the starting frequency, and *f_m_* is the frequency of the sampling point. Bring Equation (17) into Equation (18) and expand the cosine term to obtain:

(19) $$\begin{array}{l} Z_{ab}(f_{1})=e^{j2\pi f_{1}R((\cos(\theta_{b})-\cos(\theta_{a}))\cos(\theta_{0})+(\sin(\theta_{b})-\cos(\theta_{a}))\sin(\theta_{0}))/c}\\ Z_{ac}(f_{m})=e^{j2\pi f_{m}R((\cos(\theta_{c})-\cos(\theta_{a}))\cos(\theta_{0})+(\sin(\theta_{c})-\cos(\theta_{a}))\sin(\theta_{0}))/c} \end{array}$$

Let $A_{1}=\cos(\theta_{b})-\cos(\theta_{a})$ and $B_{1}=\sin(\theta_{b})-\sin(\theta_{a})$, and then take the conjugate of two cross-spectra and multiply:

(20) $$Y_{m}=Z_{ab}(f_{1})\cdot Z_{ac}{}^{*}(f_{m})$$

Let $\gamma =\arccos\frac{A_{3}}{\sqrt{A_{3}{}^{2}+B_{3}{}^{2}}}$, $A_{3}=f_{1}A_{1}-f_{m}A_{2}$, $B_{3}=f_{1}B_{1}-f_{m}B_{2}$, and Equation (20) be transformed into:

(21) $$Y_{r}=e^{j2\pi R\sqrt{A_{3}{}^{2}+B_{3}{}^{2}}\cos(\gamma -\theta_{0}))/c}$$

In order to construct a circular array manifold, i.e., $e^{j2\pi R\cos(m\theta_{s}-\theta_{0}))/c}$, where $\theta_{s}=2\pi /M$. Let $\gamma =m\theta_{s}$, Equation (21) can be obtained:

(22) $$\cos m\theta_{s}=\frac{A_{3}}{\sqrt{A_{3}{}^{2}+B_{3}{}^{2}}}$$

According to Equation (23), *f_m_* can be solved:

(23) $$f_{m}=\frac{B_{1}\cos(m\theta_{s})-A_{1}\sqrt{1-\cos^{2}(m\theta_{s})}}{B_{2}\cos(m\theta_{s})-A_{2}\sqrt{1-\cos^{2}(m\theta_{s})}}f_{1}$$

Therefore, it is only necessary to know the coordinates of the three array elements, and it is easy to obtain *R*, $\theta_{a}$, $\theta_{b}$, $\theta_{c}$ according to the geometric relationship. The target position can be estimated. Beamforming is shown in Equation (24):

(24) $$\mathrm{Beam}(\theta )=\sum_{m=1}^{M}Z_{ab}(f_{1})Z_{ac}{}^{*}(f_{m})e^{j2\pi R\sqrt{A_{3}{}^{2}+B_{3}{}^{2}}\cos(m\theta_{s}-\theta_{0}))/c}$$

In this way, we have implemented a wideband signal based on the azimuth estimates of the three hydrophones. Because the algorithm is designed with reference to the circular array, there is no problem with port and starboard ambiguity.

## 3. Simulation

### 3.1. Comparison of the Cross-Correlation Algorithm and Frequency Diversity Algorithm

**Figure 4:**
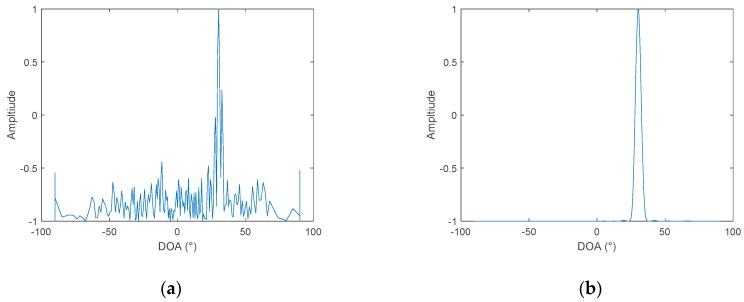
DOA of two hydrophones, SNR = 0 dB: (**a**) the cross-correlation method and (**b**) frequency diversity algorithm.

The cross-correlation method and cross-spectrum method have a similar performance under the same signal-to-noise ratio. Moreover, the conventional cross-spectral method is based on the discrete spectrum of the received signal to directly estimate the azimuth. In practical applications, the relative frequency deviation of signals and the leakage of spectrum will lead to an azimuth estimation error. Thus, here, we only compare the proposed algorithm with the cross-correlation method. Assuming that the two hydrophones are 128m apart, the signal is Gaussian white noise, with 100–200 Hz bandpass filtering. First, consider a single target with an incident angle of 40°. The sampling frequency is 4 kHz, and the number of samples is 4096. According to the relevant algorithm processing time length, set Δf=1 Hz in the frequency diversity algorithm. The sampling bandwidth MΔf is set to 100 Hz according to the signal bandwidth. When the in-band SNR of the received hydrophone signal is set to 0 dB, where the noise is Gaussian white noise, the simulation result is shown in Figure 4.

**Figure 5:**
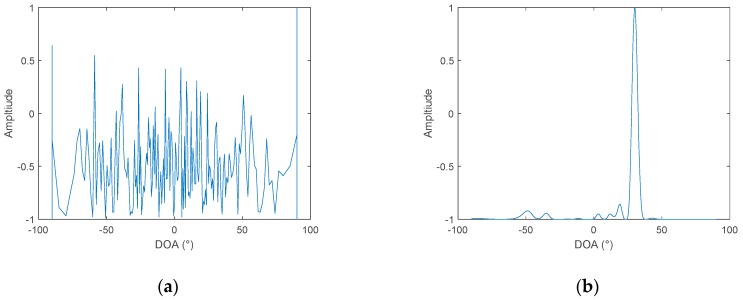
DOA of two hydrophones, SNR = −16 dB: (**a**) The cross-correlation method and (**b**) frequency diversity algorithm.

**Figure 6:**
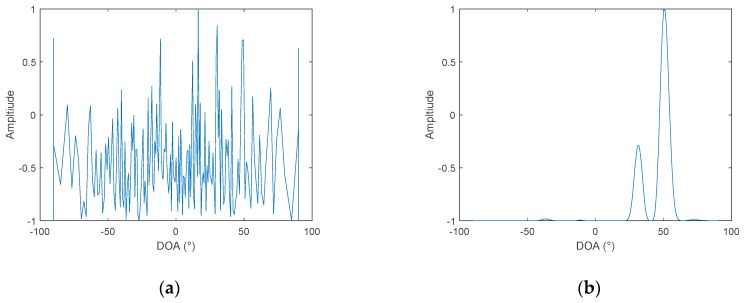
DOA of two hydrophones, SNR = −10 dB, two targets: (**a**) the cross-correlation method and (**b**) frequency diversity algorithm.

When the in-band SNR is set to −16 dB, the simulation results are shown in Figure 5:

It can be seen, from Figure 4 and Figure 5, that the DOA estimation performance of the frequency diversity algorithm is superior to the cross-correlation method under different SNR. When the SNR is reduced to −16 dB, the cross-correlation method can no longer estimate the azimuth of the target, while using the frequency diversity algorithm, and a robust estimation of the target azimuth can still be achieved. Change the number of sound sources to two, and the bearing angles are 30° and 50°. The in-band SNR is set to −10 dB. The simulation results are shown in Figure 6.

When the number of sound sources is changed to two, it can be seen from Figure 6, that when the in-band SNR is reduced to −10 dB, the cross-correlation method can no longer estimate the azimuth of the target. This is because the source signal has a wideband, and the two target signals are not completely independent. Therefore, there are many periodic pseudo peaks in the cross-correlation. Therefore, when the number of targets changes from 1 to 2, the SNR used to compare the performance of the two algorithms is increased from −16 dB to −10 dB. When frequency diversity techniques are used, the target azimuth can still be estimated robustly. The reason is that the frequency diversity technique uses the phase relationship in the signal frequency dimension, so the processing gain is improved, compared to the cross-correlation method, and the resolution is not affected by the correlation between signals. Since the simulated two target signals are band-limited white Gauss noise, whose spectrum is random, in the latter analysis, it can be found that the beamformed output amplitude obtained by the proposed algorithm, is related to the energy distribution in the frequency domain, so the amplitudes of the two sources are different.

### 3.2. Resolution of the Frequency Diversity Algorithm

**Figure 7:**
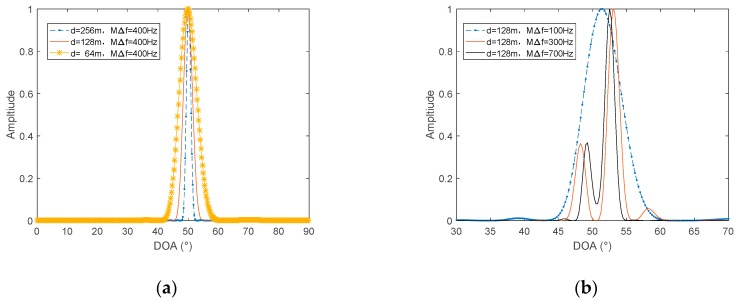
Azimuth resolution of the proposed algorithm, with different *d* and MΔf. (**a**) Sensor interval *d* and (**b**) processing bandwidth MΔf.

According to Equation (15), increasing the signal processing bandwidth MΔf and the sensor interval *d* can improve the resolution of the algorithm. Figure 7 is a simulation verification of this property. The sampling frequency is 4 kHz, the number of samples is 4096, Δf=1 Hz, and the starting frequency $f_{0}=100\;\mathrm{Hz}$.

In Figure 7a, there is only one target at 50°. When the processing bandwidth MΔf is set to 400 Hz, as the sensor interval decreases, the width of the main lobe becomes wider, so the resolution decreases. The two targets in Figure 7b have an incoming wave direction of 50° and 52°. When *d* is set to 128 m, as the processing bandwidth MΔf increases, the main lobe width becomes narrower, and the resolution increases. In addition, when MΔf=100 Hz, the algorithm cannot separate two targets. When MΔf=300 Hz, the algorithm can separate the two targets, but the peaks do not appear exactly at 50° and 52°, but at 48° and 53°. When MΔf=700 Hz, the two peaks appear at exactly 50° and 52°, so the azimuth estimation is accurate. In summary, the larger the sensor interval, the wider the processing bandwidth MΔf, the higher the resolution of the algorithm, and the more accurate the azimuth estimation.

### 3.3. Simulation of Frequency Diversity Based on Three Hydrophones

**Figure 8:**
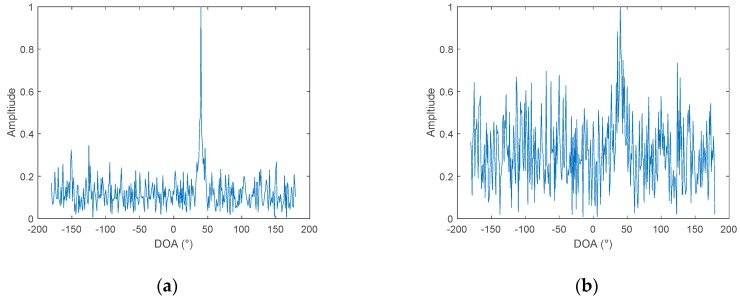
DOA estimation results under different SNR: (**a**) SNR = 0 dB (**b**) SNR = −10 dB.

Assuming three hydrophones, the positions of which are not in a straight line, the coordinates are: (0,0), (−325 m, 141 m), (−253 m, −213 m). The radius of the circle is determined to be 202 m, according to the position of the three points. The target signal is 50–1000 Hz band-limited white noise, and the incident angle is 40°. Using Equation (23) to calculate *f*_m_ according to *f*_1_, wherein the sampling point *M* is set to 512, the azimuth estimate can be obtained according to Equation (24). The result is shown in Figure 8.

**Figure 9:**
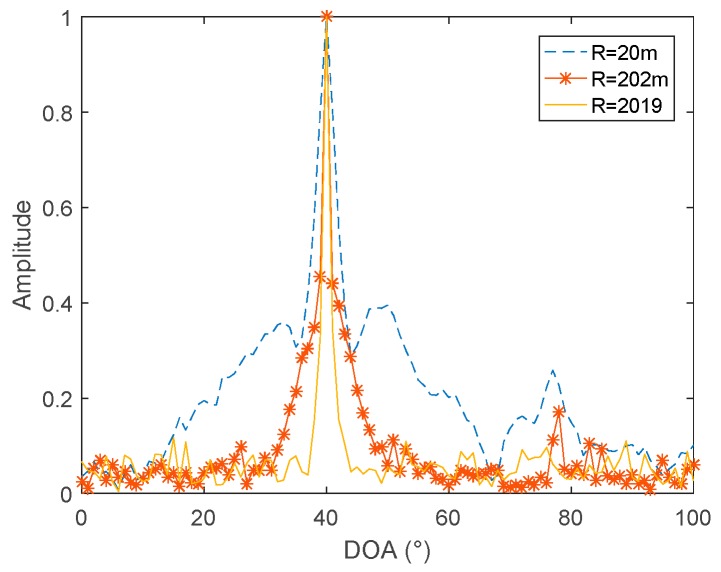
The relationship between the azimuth resolution and the virtual radii of three hydrophones.

From Figure 8, it can be seen that, when three hydrophones are used, an azimuth estimation result of −180° to 180° can be obtained, and there is no problem with port and starboard ambiguity. When the position of the three hydrophones changes, the radius of the virtual circle changes. The simulations compared the azimuth estimation results under the three apertures, with radii of 20 m, 202 m, and 2019 m, as can be seen in Figure 9:

As can be seen, in Figure 9, the increase in the radius of the virtual circle is beneficial to the resolution. The farther the distance is placed in the three hydrophones, the better the azimuth estimation performance.

## 4. Experimental Data Verification

The algorithm is first verified with Swell 96 horizontal south array data [21], using the 14th to 28th array elements. The SWellEx-96 Experiment was conducted between May 10 and 18, 1996, approximately 12 km from the tip of Point Loma near San Diego, California. Acoustic sources, towed from the R/V Sproul, transmitted various broadband and multi-tone signals at frequencies between 50 and 400 Hz.

**Figure 10:**
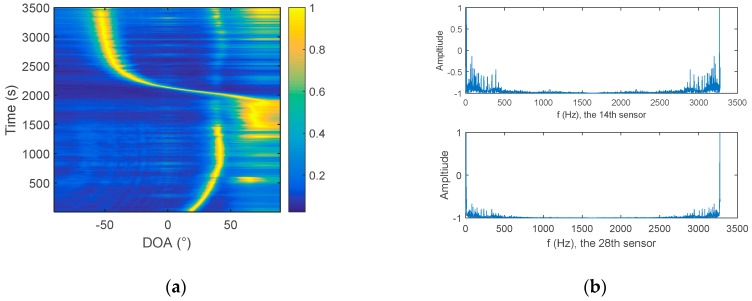
(**a**) Azimuth history diagram of Swell 96 data. (**b**) Signal spectrum of the 14th and 28th sensors.

In order to further compare the performance of the two hydrophone algorithms, conventional array processing is used to obtain the azimuth estimation result as a reference, because the array gain of the processing of multiple array elements leads to a clear trajectory. The processing frequency bandwidth is 20–1000 Hz. The azimuth history diagram is shown in Figure 10a. It can be seen, from the figure, that within this time period (1–3500 s), there are mainly two targets, one with a large span in the azimuth, and one mainly at around 40°.

**Figure 11:**
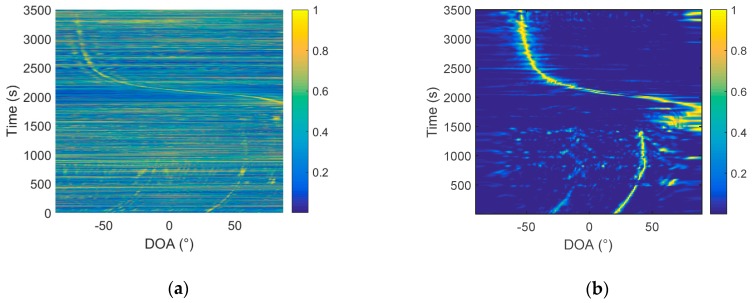
Azimuth history diagram of the two hydrophones: (**a**) The cross–correlation method and (**b**) frequency diversity algorithm.

The 14th array element and the 28th array element are selected as the two hydrophones, and the distance between them is 106 m. Figure 10b is the signal spectrum of the 14th and 28th elements, and the Fourier transform time is from 1000 s to 1001 s. The results of the cross-correlation method and the frequency diversity algorithm are shown in Figure 11. The sampling frequency *f*_s_ is 3277 Hz, the number of samples is 3277, and Δf=1 Hz. The processing bandwidth MΔf is set to 980 Hz, according to the processing bandwidth of 20–1000 Hz.

**Figure 12:**
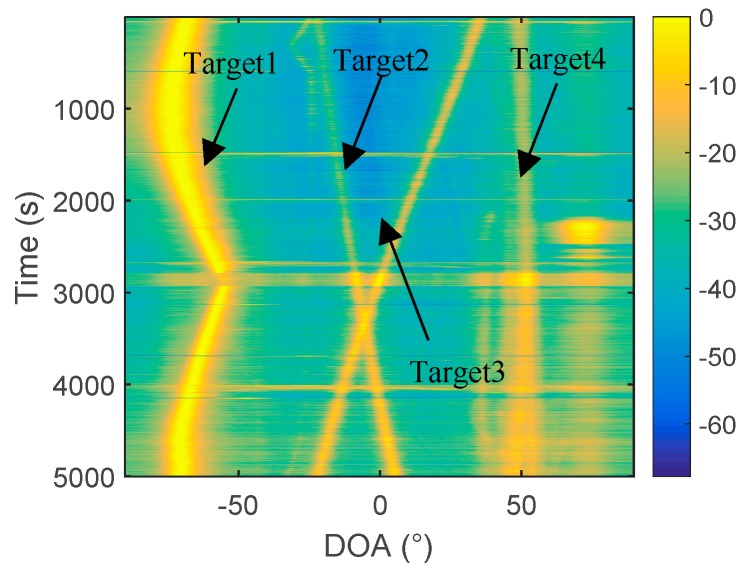
Azimuth history diagram of the 64-element array.

From the comparison in Figure 11, it can be found that, using the same processing time, the same hydrophone, the target trajectory, estimated by the frequency diversity algorithm, is obviously clearer than that obtained by the cross-correlation algorithm. The algorithm is further verified by the South Sea data. Similarly, the conventional array processing is performed first, and a relatively accurate orientation estimation result is obtained. Then, we compare the cross-correlation method based on the passive two-hydrophone and the frequency diversity algorithm. In the array processing, 64 array elements are selected, with an interval of 4 m, and the processing method uses CBF. The processing frequency band is 20–400 Hz, and the sampling frequency is 2048 Hz. The azimuth estimation results are in Figure 12.

**Figure 13:**
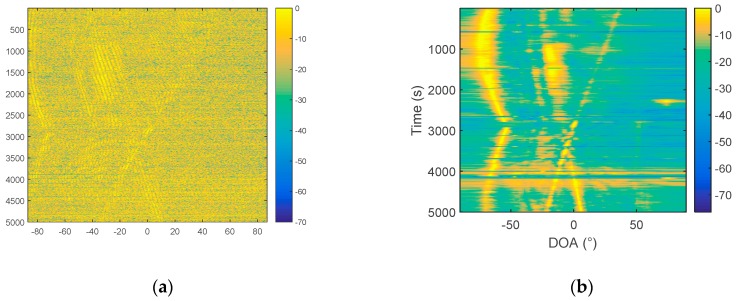
Azimuth history diagram of the two hydrophones: (**a**) the cross-correlation method and (**b**) frequency diversity algorithm.

The data are processed using two hydrophones, as shown in Figure 13, and the number of samples is 2048, Δf=1 Hz, and MΔf is set to 380 Hz.

**Figure 14:**
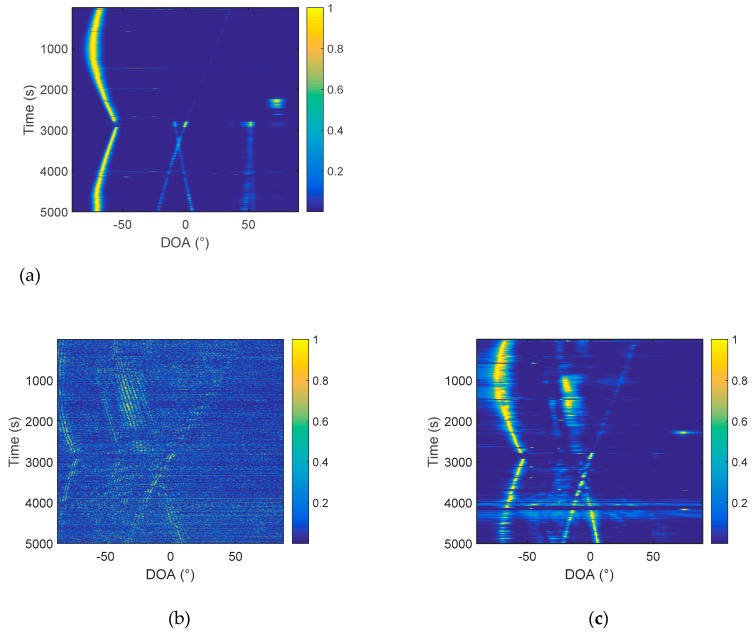
Azimuth history diagram of the two hydrophones: (**a**) The conventional 64-element processing; (**b**) two hydrophones, cross-correlation algorithm; and (**c**) two hydrophones, frequency diversity algorithm.

In Figure 13a, only a little blurred outline can be seen, and the trajectory of the target can hardly be observed. In Figure 13b, the trajectories of target 1, target 2, and target 3 can be clearly observed. The trajectory of target 4 is not clear. It can be explained that the performance of the two-hydrophone algorithm based on frequency diversity technology is significantly higher than that of the cross-correlation algorithm. Moreover, we found, in the experiment, that the frequency diversity algorithm has a higher processing gain, which is easily seen before taking the beam energy by 10 lg. Before taking 10 lg, the beam energy is shown in Figure 14.

**Figure 15:**
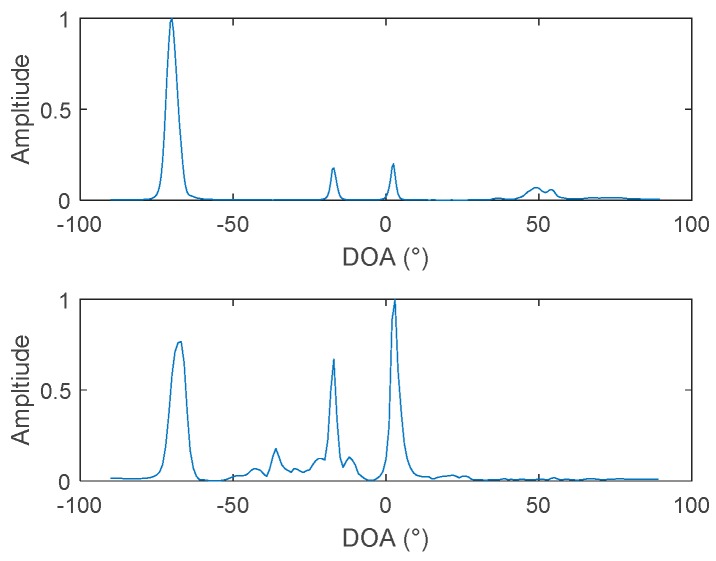
DOA results at 4500s: (**top**): 64-element array processing, and (**bottom**): the two-hydrophone frequency diversity algorithm.

Comparing Figure 14b with Figure 14c, it is found that the frequency diversity algorithm is better than the cross-correlation algorithm, regardless of whether the log is taken or not. Furthermore, comparing Figure 14c with Figure 14a, it can be found the energy of target 2 and target 3 is significantly improved when the frequency diversity algorithm is used. To further reflect this feature, we take the azimuth estimation result at 4500 s as an example. At this time, target 2 and target 3 are located at 3° and −17°, respectively, and it is apparent, from the comparison of Figure 15, that the energy of target 2 and target 3 is enhanced.

**Figure 16:**
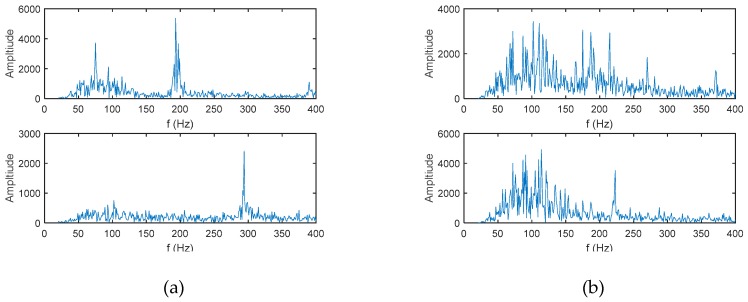
Frequency spectrum of the four targets. (**a**) The upper picture is target 1, and the lower picture is target 4; and (**b**) the upper picture is target 2, and the lower picture is target 3.

The reason for this phenomenon is that the frequency diversity algorithm of the two hydrophones mainly uses the frequency domain information of the signal. Therefore, rich frequency domain information and uniform frequency domain energy distribution are beneficial for the energy of the beamforming output. The spectrums of Target 1 and Target 4 are shown in Figure 16a, and the spectra of target 2 and target 3 are shown in Figure 16b. From the comparison of Figure 16a,b, the spectrums of target 2 and target 3 are significantly richer than that of target 1 and target 4, and the energy distributions are more uniform. Therefore, in the estimation results of the two hydrophones, the energy of targets 2 and 3 is strengthened. Among them, the spectrum energy distribution of target 4 is the most concentrated so, in the two-hydrophone azimuth estimation, target 4 can hardly be observed.

## 5. Conclusions

This paper proposes a frequency diversity algorithm to achieve passive azimuth estimation using two hydrophones. Compared with the traditional cross-correlation method, the algorithm has a high processing gain and can obtain a clear target trajectory. When the energy of the target signal is evenly distributed in the frequency domain, the processing gain of the algorithm using two hydrophones may even exceed the CBF processing gain of multiple array elements. In addition, in the theoretical derivation, the relationship between the resolution, the sensor interval and the processing bandwidth is analyzed. In the simulation and experiment, the feasibility of the algorithm and the advantages, compared with the cross-correlation method, are verified.

The algorithm proposed in this paper can obtain a clear target trajectory using the wideband signals received by only two hydrophones. This is significant for the application of target estimation in the field of buoy and AUV collaborative operations. The idea of using frequency domain information to virtualize array element domain information has important academic value for passive sonar azimuth estimation.
